# Post-Transplant Double Malignancy: Multiple Myeloma and Papillary Renal Cell Carcinoma—A Case Report

**DOI:** 10.3390/reports9010013

**Published:** 2025-12-30

**Authors:** Aleksandar Petrov, Miroslava Benkova, Yavor Petrov, Yana Dimieva, Mari Ara Hachmeriyan

**Affiliations:** 1 Clinic of Nephrology, University Hospital “St. Marina” EAD, 1 Hristo Smirnenski Blvd., 9010 Varna, Bulgaria; 2 Clinic of Hematology, University Hospital “St. Marina” EAD, 1 Hristo Smirnenski Blvd., 9010 Varna, Bulgaria; 3 Laboratory of Medical Genetics, University Hospital “St. Marina” EAD, 1 Hristo Smirnenski Blvd., 9010 Varna, Bulgaria

**Keywords:** kidney transplantation, post-transplant malignancy, multiple myeloma, papillary renal cell carcinoma, native kidney, tacrolimus, VCd, DKd, case report

## Abstract

**Background and Clinical Significance:** Kidney transplant recipients have a 2–4-fold higher cancer risk than the general population. The sequential occurrence of multiple myeloma (MM) and native-kidney renal cell carcinoma (RCC) is rare and creates competing priorities between anti-myeloma efficacy and allograft preservation. **Case Presentation:** A 54-year-old woman with a 2020 living-donor kidney transplant presented in 2024 with bone pain and shoulder swelling. Low-dose whole-body CT showed multiple punched-out osteolytic lesions. Work-up revealed IgG-κ M-protein 38.5 g/L and 25% clonal plasma cells; cytogenetics showed a complex karyotype (R-ISS III). First-line bortezomib/cyclophosphamide/dexamethasone (VCd) was given while maintaining tacrolimus plus low-dose steroid. After four cycles, she achieved very good partial response (M-protein 42.3 to 5.6 g/L) with stable graft function. Follow-up imaging detected a large exophytic mass in the native right kidney; nephrectomy confirmed papillary RCC, type II. Later, the myeloma progressed with epidural extension causing cord compression. Second-line daratumumab/carfilzomib/dexamethasone (DKd) and palliative spine radiotherapy were initiated. The course was complicated by opportunistic infection and pancytopenia, and the patient died in January 2025. **Conclusions:** Vigilant post-transplant cancer surveillance—including native-kidney RCC—tailored immunosuppression, and multidisciplinary coordination are critical. VCd with tacrolimus may be feasible when graft preservation is prioritized; however, relapsed high-risk MM on DKd carries substantial infectious risk and a guarded prognosis.

## 1. Introduction and Clinical Significance

Chronic kidney disease (CKD) is a major global health problem, associated with high mortality, disability, and a disproportionate impact on vulnerable populations. Widespread shortcomings in screening programs and the “silent” course of kidney diseases lead to a rapidly increasing prevalence of end-stage kidney disease (ESRD), and consequently to an ever-growing burden on health and social systems [1]. Among renal replacement options—hemodialysis, peritoneal dialysis, and kidney transplantation—transplantation offers the greatest chance for return to work capacity, social reintegration, and long-term survival, outperforming dialysis both in quality of life and with lower cardiovascular mortality [2]. Extracts from the Global Observatory on Donation and Transplantation (GODT) registers attest to the scale of this therapy: in 2023, 172,409 solid organ transplants were performed worldwide, of which 111,135 were kidney transplants [3]. A combination of refined surgical techniques and intensive advances in immunosuppressive regimens has determined the high 5-year survival rate among kidney transplant recipients—exceeding 90% [4]. Yet, this therapeutic progress brings to the forefront the delayed risks associated with transplantation—cardiovascular complications, infections, and, especially, the development of malignancies.

Data show nearly a 20% increase in the risk of post-transplant malignancies after the 10th year [5], with the percentage continuing to rise thereafter. In addition, post-transplant cancers have become the second most common cause of mortality after cardiovascular complications [6,7,8]. In a retrospective study of a Dutch cohort of 12,805 kidney transplant recipients over ~40 years, malignancies occurred in 7.1% and were associated with significantly shorter survival, even though 81% of deaths occurred with preserved graft function.

Although transplant recipients have a two- to four-fold higher risk of developing malignant neoplasms compared with the general population [9], specific differences in the tumor types that occur are also observed. Recipients less commonly develop cancers typically frequent in the general population, such as lung, colorectal, and breast cancer [10], whereas neoplasms associated with oncogenic viruses—such as non-Hodgkin lymphomas and Kaposi sarcoma—show dramatically increased incidence [11,12,13].

Among the most common post-transplant lymphoproliferative disorders (PTLDs), rarer hematologic neoplasms such as multiple myeloma (MM) have also been described [14]. Multiple myeloma is a malignant plasma cell disorder characterized by clonal proliferation of plasma cells in the bone marrow and secretion of monoclonal immunoglobulins and/or light chains, leading to organ damage including bone destruction, kidney injury, anemia, and immune dysfunction. According to the updated diagnostic criteria of the International Myeloma Working Group (IMWG), diagnosis requires ≥10% clonal plasma cells in the bone marrow or biopsy-proven plasmacytoma, plus the presence of “myeloma-defining events,” such as the CRAB criteria (hypercalcemia, renal impairment, anemia, bone lesions) or SLiM criteria (≥60% bone marrow plasma cells, serum free light chain ratio ≥ 100, or ≥1 focal lesion on MRI) [15]. In the general population, MM accounts for approximately 10% of malignant hematological diseases, with a median age at diagnosis of 69 years [16]. Over the past two decades, the incorporation of proteasome inhibitors, immunomodulatory agents, and monoclonal and bispecific antibodies, as well as improved supportive care, has significantly extended overall and progression-free survival. Nevertheless, the disease remains incurable for most patients, and data on the occurrence of this plasma cell dyscrasia among kidney transplant recipients are scarce.

Of further interest is monoclonal gammopathy of undetermined significance (MGUS), considered a precursor condition to MM. MGUS is defined by the presence of a small amount of monoclonal (“M”) protein in serum (usually <30 g/L) and a clonal plasma cell population in the bone marrow (<10%) without organ damage or disease-related symptoms [17]. Although MGUS is asymptomatic and often detected incidentally, serial studies report cumulative progression of ~12% at 10 years, 25% at 20 years, and up to 30% at 25 years (a roughly constant ~1% annual risk) [18]. Advanced age is a risk factor for MGUS, and, as indications for transplantation have expanded to older patients, MGUS is increasingly encountered before or after transplantation [19,20]. However, MGUS is not considered a contraindication to transplantation, as prospective studies show that the rate of malignant evolution of MGUS in transplant recipients is comparable to that in the general population [21].

Beyond hematologic malignancies, kidney transplant recipients carry a particularly high risk of certain solid tumors such as renal cell carcinoma (RCC), most often in the native kidneys. Registry data from the United Kingdom show a 15-fold increased risk of RCC compared with non-transplanted controls [22]. Similarly, a large multicenter Italian cohort reported a significant excess of de novo neoplasia, with RCC among the most frequent solid tumors [23]. Prolonged immunosuppression and impaired immune function are not the only factors predisposing to RCC. Specific renal factors—such as prolonged pre-transplant dialysis [24], acquired cystic kidney disease (ACKD), and persistent native kidneys after transplantation—contribute to post-transplant tumor development. In practice, the vast majority of kidney carcinomas arise in the native kidneys rather than the graft—over 90% of RCCs in kidney recipients are of native origin [25]. The risk is especially high with marked ACKD during dialysis. By contrast, RCC arising in the allograft itself is rarer and may be due to donor transmission (a small, undetected tumor in the donor kidney growing under immunosuppression) or develop de novo years later.

The concomitant presence of MM and RCC in kidney transplant recipients, however, is extraordinarily rare in the literature and poses specific diagnostic and therapeutic challenges. Here, we present the case of a 54-year-old woman—the recipient of a kidney transplant—who developed multiple myeloma followed by papillary RCC, and we emphasize the clinical course, therapeutic dilemmas, and a review of the relevant literature.

## 2. Case Presentation

This concerns a 54-year-old woman, A Rh(+), who, on the basis of long-standing arterial hypertension, developed CKD, detected incidentally after a subsequent hypertensive crisis in June 2017. In May 2019, she was hospitalized at University Hospital “St. Marina”, Varna with edema and a worsened kidney function; given bilateral ultrasound nephrosclerosis, a kidney biopsy was not performed (hypertensive nephropathy and a latent glomerulonephritis were considered in the differential). The patient then discontinued follow-up in the clinic, and, due to progression to ESRD in April 2020, started peritoneal dialysis; two months later, she underwent kidney transplantation from a living unrelated donor (husband, 0 Rh+). The transplantation was performed in a hospital outside the country and we do not have a complete medical report for the HLA match, etc. During post-transplant follow-up through 2022, no significant clinical issues were identified.

**Figure 1:**
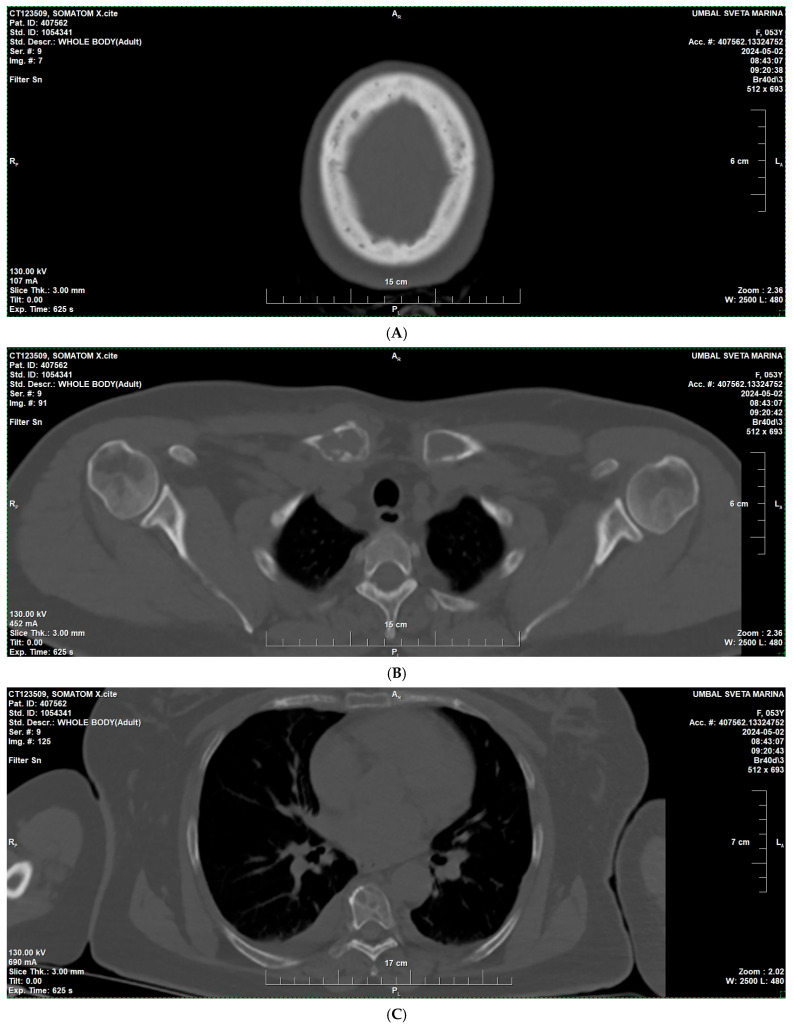

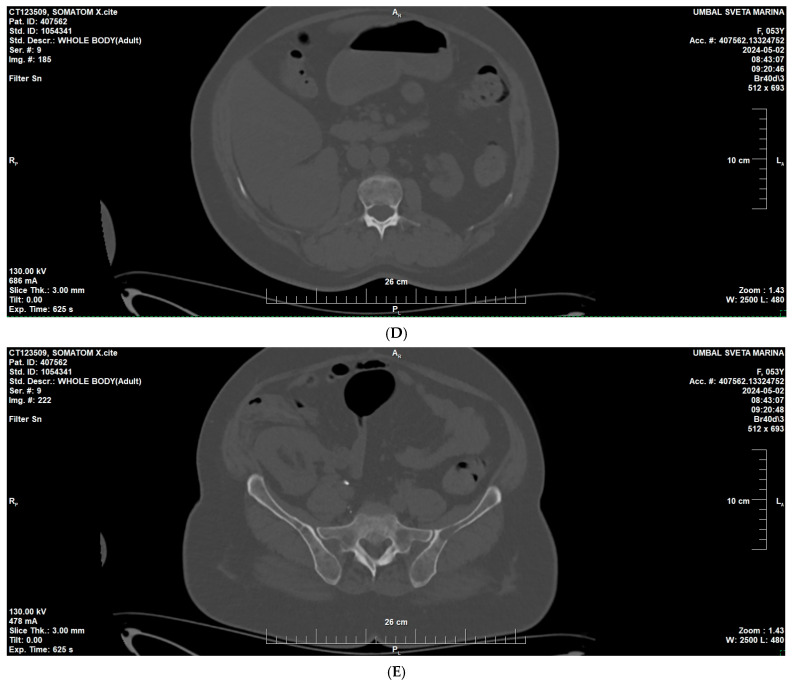
Low-dose whole-body CT findings at diagnosis of multiple myeloma—multiple osteolytic lesions. (**A**) Skull CT showing multiple “punched-out” osteolytic lesions in the calvaria. (**B**) CT of the right clavicle demonstrating an expansile osteolytic lesion with cortical thinning. (**C**) CT of the thorax and lumbal region (**D**) showing osteolytic lesions involving the vertebral bodies and ribs. (**E**) CT of the pelvis demonstrating multiple osteolytic foci in the iliac and pubic bones.

In April 2024, due to increasing bone pain and a swelling in the right shoulder area, a low-dose whole-body CT was performed, revealing multiple osteolytic lesions without a sclerotic rim in the bones of the calvaria and mandible (A); in the bodies and arches of the cervical (B), thoracic (C), and lumbar vertebrae (D); ribs; sternum; iliac (E) and pubic bones, with some lesions showing osseous expansion—in the right clavicle and scapula (Figure 1).

She was referred to the hematology clinic for diagnostic work-up and treatment. Laboratory tests showed a monoclonal IgG κ paraprotein of 38.5 g/L on serum protein electrophoresis, an abnormal free light chain ratio (κ 24.88 mg/L, λ 11.12 mg/L, κ/λ = 2.24), and hypogammaglobulinemia of uninvolved immunoglobulins. Bone marrow analysis showed 25% plasma cell infiltration with preserved trilineage hematopoiesis. Cytogenetic analysis revealed a complex karyotype with numerical and structural abnormalities (40,X,i(1)(q10),del(1)(q32),-4,-10,-10,-13,-14,-22,+mar [12]/46, XX [8]); FISH for del(17p) (TP53 locus) was negative. Based on these data, multiple myeloma, IgG κ type, R-ISS stage III was diagnosed.

At a multidisciplinary board, we discussed management given suspected myeloma nephropathy and the need for histologic verification; during an attempted core biopsy of the transplant, marked hemorrhagic diathesis occurred with a normal coagulation profile, and the procedure was halted. The morphological material obtained was non-diagnostic (absence of glomeruli; tubular degenerative changes without atrophy; interstitium without fibrosis/lymphoid infiltrates; subintimal fibrosis of a small artery; immunofluorescence negative for IgG/IgA/IgM/C3/C4c/C1q/C4d/fibrinogen/κ/λ), without convincing evidence for myeloma cast nephropathy. First-line therapy with bortezomib, cyclophosphamide, and dexamethasone (VCd) was initiated. It was decided to keep graft immunosuppression calcineurin-inhibitor-based with tacrolimus (Envarsus), target 5–7 ng/mL, plus low-dose corticosteroid (methylprednisolone 10 mg). A switch to an mTOR inhibitor was considered but tacrolimus was prioritized. After four cycles, the patient achieved a very good partial response (VGPR) with a marked decrease in paraprotein and clinical improvement.

**Figure 2:**
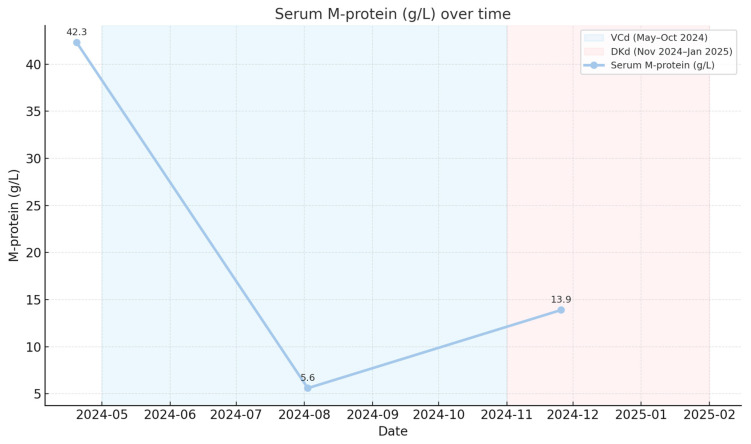
Dynamics of serum monoclonal protein (M-protein) during therapy.

Serum M-protein decreased from 42.3 g/L (19 April 2024) to 5.6 g/L (2 August 2024) following initiation of VCd therapy, corresponding to a very good partial response. Biochemical progression was documented in November 2024 prior to the initiation of DKd (Figure 2).

**Figure 3:**
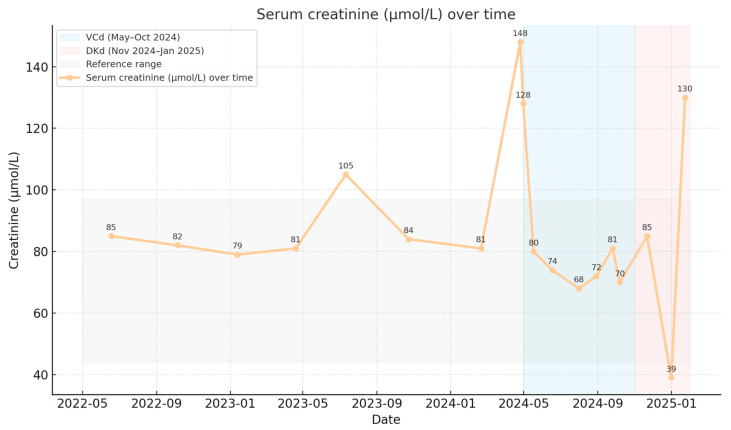
Renal allograft function over time.

Serum creatinine remained stable from May 2022 to January 2025 without a statistically significant linear trend (slope −0.79 μmol/L per month; *p* = 0.091). Median creatinine values were 85 μmol/L prior to therapy, 76 μmol/L during VCd, and 78.5 μmol/L during DKd (Figure 3).

**Figure 4:**
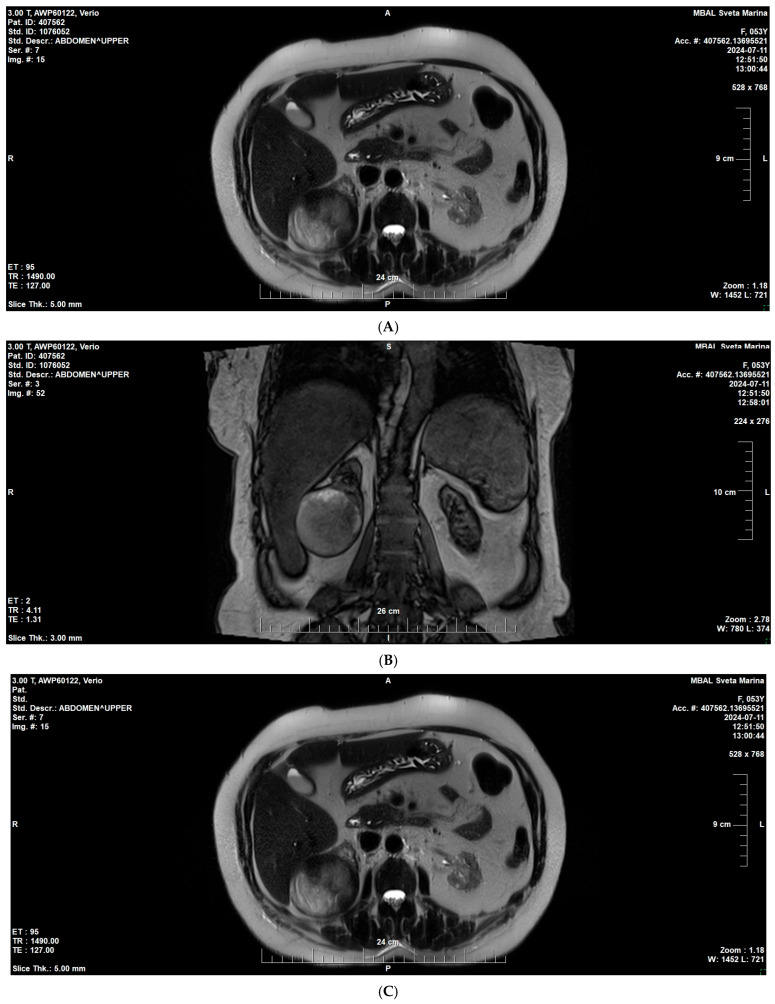
MRI of the native right kidney (July 2024). (**A**) Coronal T1-weighted image showing a well-circumscribed exophytic mass in the lower pole of the right native kidney (59 × 67 × 68 mm). (**B**,**C**) Axial T2-weighted images demonstrating heterogeneous signal intensity.

On follow-up imaging, a suspicious lesion was identified in the lower pole of the right native kidney measuring 59 × 67 × 68 mm. Subsequent right nephrectomy and histological analysis demonstrated papillary RCC, type II (Figure 4 A–C).

Subsequent nephrectomy confirmed papillary renal cell carcinoma, type II.

**Figure 5:**
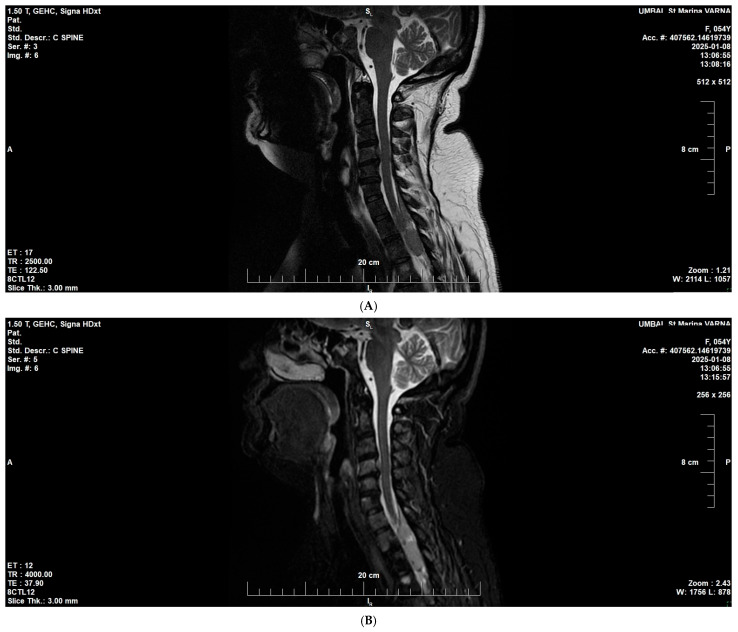
Cervicothoracic MRI at disease progression (January 2025). (**A**) Sagittal T2-weighted image showing diffuse bone marrow infiltration consistent with multiple myeloma. (**B**) Sagittal T2/STIR image demonstrating epidural soft-tissue extension at C4–C5 and C7–T2, causing thecal sac compression at T2–T3, without radiological evidence of myelopathy.

At the end of 2024, the patient demonstrated progression of the hematologic disease with new extramedullary lesions and a newly emerged neurologic deficit due to spinal cord compression in the cervical and thoracic segments (assessed as inoperable). Follow-up MRI showed diffuse infiltration of the vertebral bone marrow spaces compatible with multiple myeloma, with epidural extension at levels C4–C5 and C7-Th2. The lesion caused compression of the thecal sac at Th2–Th3, without radiologic signs of spinal cord myelopathy (Figure 5A,B).

Second-line therapy with the anti-CD38 monoclonal antibody daratumumab, second-generation proteasome inhibitor carfilzomib, and dexamethasone (DKd) was initiated, in parallel with palliative radiotherapy to thoracic vertebral lesions—8 Gy per fraction to a total of 16 Gy every other day using IMRT, 6-MV photons. Despite initial stabilization, treatment was complicated by acute infections, including Candida albicans pneumonia and colonization with multidrug-resistant Pseudomonas aeruginosa. Laboratory monitoring showed progressive pancytopenia, persistent anemia (Hb 74–91 g/L), thrombocytopenia (to 49 × 10^9^/L), and hypogammaglobulinemia. Despite supportive measures—broad-spectrum antibiotics, antifungals, transfusion therapy, and dose-adjusted immunosuppression—the patient’s condition deteriorated. On 23 January 2025, she developed acute circulatory failure and death was confirmed.

## 3. Discussion

### 3.1. Multiple Myeloma and RCC in Transplant Recipients

Over the past two decades, post-transplant malignancies have become one of the leading causes of morbidity and mortality in solid organ recipients. Registry studies show an overall cancer risk approximately 2–4 times higher than in the general population, often due to long-term immunosuppression and recurrent viral infections that compromise anti-tumor immune surveillance. In this context, our case—MM and RCC after kidney transplantation—is exceptionally rare. Based on the series by Choueiri et al. of 1100 myeloma patients and 2704 with RCC (non-transplanted), only eight individuals developed both diseases; four were first diagnosed with myeloma followed by RCC 3–46 months later, and four in the reverse sequence (1–108 months) [26], while, among transplant recipients, the publications describing such cases are isolated. Singh N. and colleagues review plasma cell dyscrasias after solid organ transplantation and emphasize that, although MM is uncommon, it is associated with aggressive biology and unfavorable outcomes in transplant recipients [27,28]. Only individual clinical cases have been published, suggesting possible shared pathogenetic mechanisms or coincidental occurrence [29,30].

### 3.2. Diagnostic Aspects

In kidney recipients, the differential diagnosis includes distinguishing MM from other post-transplant lymphoproliferative disorders (PTLDs) with plasmacytic features. In this patient, bone marrow morphology and flow-cytometry were consistent with a clonal plasma cell neoplasm. Allograft biopsy (albeit scant) did not categorically confirm myeloma cast nephropathy or acute rejection, supporting preserved graft integrity.

### 3.3. Genetic Profile of the Malignancies

The cytogenetic analysis performed—although part of a limited panel relative to the latest risk-stratification recommendations [31]—is crucial. The identified complex karyotype is one of the strongest markers of high risk and poor prognosis in MM. Although the absence of deletion of the TP53 tumor-suppressor gene is considered favorable, its significance here is minimal against the backdrop of multiple other high-risk aberrations. This genetic profile explains the aggressive biology—rapid progression, extramedullary disease, and suboptimal therapeutic response.

Regarding papillary RCC, recent years have brought a revolutionary change in understanding of the disease. The 2022 WHO classification of urinary and male genital tumors (5th edition) is fundamentally molecular and contains major revisions compared with 2016 [32]. Diagnosis is no longer based solely on histology (such as type I and II) but on specific genetic alterations. In this context, next-generation sequencing (NGS) is increasingly recommended and implemented in routine practice, allowing precise classification and discovery of potential targets. Access, however, remains uneven, and in this patient such analysis was not performed due to financial constraints. Such tumor profiling could offer key insights—identifying particular somatic mutations (e.g., in VHL, FH, or BAP1) may prompt testing for the same mutation in germline DNA.

This possibility is especially important because the coincident emergence of two rare and unrelated malignancies raises the hypothesis of a shared etiology beyond immunosuppression. Although the combination of MM and RCC is not characteristic of known tumor predisposition syndromes (e.g., von Hippel–Lindau, hereditary leiomyomatosis and RCC, Birt–Hogg–Dubé, BAP1 tumor predisposition), a germline mutation in a DNA-repair or tumor-suppressor gene cannot be excluded. Such a mutation could create a general oncologic predisposition, with chronic immunosuppression acting as a “second hit” permitting clinical manifestation of both neoplasms. This concept is supported by the literature showing a higher-than-expected co-occurrence of the two diseases [33,34,35].

### 3.4. Bone Marrow and Spine Involvement in Multiple Myeloma

MRI in our patient demonstrated diffuse infiltration of vertebral marrow spaces consistent with advanced MM, with epidural spread at C4–C5 and C7-Th2. At Th2–Th3, there was thecal sac compression without radiologic evidence of myelopathy. Spinal involvement in MM is well-described: approximately 15–20% of patients develop epidural disease, often leading to spinal cord compression [36]. Early recognition is critical, as timely therapy (radiotherapy, corticosteroids, or systemic treatment) can prevent permanent neurologic deficits [37]. Diffuse infiltration, as opposed to focal lesions, is associated with more aggressive biology and worse prognosis [38]. The absence of myelopathy at presentation here allowed early intervention before irreversible damage.

### 3.5. Therapeutic Considerations

Treatment of MM in kidney transplant recipients requires a balance between anti-myeloma efficacy and preservation of allograft function. Bortezomib-based regimens are considered effective and safe in transplant recipients; bortezomib may even favorably impact antibody-mediated rejection, making it a rational first-line choice [39]. The second-line choice of daratumumab, carfilzomib, and dexamethasone (DKd) is supported by clinical trials in relapsed/refractory MM [40]. However, daratumumab is associated with hypogammaglobulinemia and higher infectious risk, and carfilzomib carries risks of cardio- and nephrotoxicity—factors complicating therapy in transplant patients.

Regarding post-transplant immunosuppression, in our case, we maintained a CNI-based regimen with tacrolimus (target 5–7 ng/mL) plus low-dose corticosteroid, despite the oncologic setting; a switch to an mTOR inhibitor was discussed but ultimately not preferred. Data from large cohorts show that mTOR inhibitors have antineoplastic effects and may reduce the risk of new cancers and improve outcomes in existing neoplasms, including post-transplant; however, real-world use remains limited and frequently discontinued due to adverse effects, making individualized selection for conversion crucial [41,42]. In a study by Atalah et al. [8], a non-definitive trend toward higher malignancy rates with lower HLA matching (as in unrelated donation) was observed, potentially an indirect marker of need for more intensive immunosuppression—though further data are required [43]. The same author notes a dual role of calcineurin inhibitors—both T-cell suppression and possible direct antitumor effects [44]. In our patient—with active high-risk MM, frequent infections during antineoplastic therapy, a recent native-kidney nephrectomy wound, and preserved graft function throughout—retaining tacrolimus was preferred as a compromise between oncologic control and limiting additional complications.

### 3.6. Prognosis and Outcome

Despite initial VGPR after VCd, the patient progressed rapidly, reflecting the aggressive profile of MM in kidney recipients. The combination of ISS stage III and complex karyotype, extramedullary disease, and infectious complications, along with the emergence of an additional solid tumor, contributed to the unfavorable outcome.

## 4. Conclusions

This case highlights the complexity of treating multiple myeloma in kidney transplant recipients. Diagnosis requires distinction from classic PTLD, careful graft assessment, and comprehensive staging. Therapy should be adapted to balance anti-myeloma efficacy with allograft preservation and control of infectious complications. The emergence of a secondary malignancy such as RCC underscores the need for stringent oncologic vigilance in transplant patients. Multidisciplinary collaboration is essential in such cases, enabling the development of tailored diagnostic and therapeutic algorithms to reduce risks in this highly vulnerable population.

## Data Availability

The original contributions presented in this study are included in the article. Further inquiries can be directed to the corresponding author.
